# Comparative analysis of statistical and deep learning-based multi-omics integration for breast cancer subtype classification

**DOI:** 10.1186/s12967-025-06662-5

**Published:** 2025-07-01

**Authors:** Mahmoud M. Omran, Mohamed Emam, Mariam Gamaleldin, Asmaa M. Abushady, Mustafa A. Elattar, Mohamed El-Hadidi

**Affiliations:** 1 Bioinformatics Group, Center for Informatics Science (CIS), School of Information Technology and Computer Science (ITCS), Nile University, Giza, Egypt; 2 School of Information Technology and Computer Science, Nile University, Giza, Egypt; 3 School of Biotechnology, Nile University, Giza, Egypt; 4 CIIMAR/CIMAR, Interdisciplinary Centre of Marine and Environmental Research, University of Porto, Terminal de Cruzeiros Do Porto de Leixões, Av. General Norton de Matos, S/N, 4450-208 Porto, Portugal; 5 Department of Biology, Faculty of Sciences, University of Porto, Rua Do Campo Alegre, 4169-007 Porto, Portugal; 6 Medical Imaging and Image Processing Research Group, Center for Informatics Science, Nile University, Giza, Egypt; 7 Present Address: Department of Cancer and Genomic Sciences, School of Medical Sciences, College of Medicine and Health, University of Birmingham Dubai, Dubai, United Arab Emirates

**Keywords:** Breast cancer, Multi-omics integration, MOFA+, MoGCN, F1 score, Network analysis, Fc gamma R-mediated phagocytosis, SNARE pathway, Personalized Medicine

## Abstract

**Background:**

Breast cancer (BC) is a critical cause of cancer-related death globally. The heterogeneity of BC subtypes poses challenges in understanding molecular mechanisms, early diagnosis, and disease management. Recent studies suggest that integrating multi-omics layers can significantly enhance BC subtype identification. However, evaluating different multi-omics integration methods for BC subtyping remains ambiguous.

**Methods:**

In this study, we conducted a multi-omics integration analysis on 960 BC patient samples, incorporating three omics layers: Host transcriptomics, epigenomics, and shotgun microbiome. We compared two integration approaches the statistical-based approach (MOFA+) and a deep learning-based approach (MOGCN) for this integration. We evaluated both methods using complementary evaluation criteria. First, we assessed the ability of selected features to discriminate between BC subtypes using both linear and nonlinear classification models. Second, we analyzed the biological relevance of the selected features to key BC pathways, focusing on transcriptomics-driven insights.

**Results:**

Our results showed that MOFA+ outperformed MOGCN in feature selection, achieving the highest F1 score (0.75) in the nonlinear classification model, with MOFA+ also identifying 121 relevant pathways compared to 100 from MOGCN. Notably, one of the key pathways Fc gamma R-mediated phagocytosis and the SNARE pathway was implicated, offering insights into immune responses and tumor progression.

**Conclusion:**

These findings suggest that MOFA+ is a more effective unsupervised tool for feature selection in BC subtyping. Our study underscores the potential of multi-omics integration to improve BC subtype prediction and provides critical insights for advancing personalized medicine in BC.

**Supplementary Information:**

The online version contains supplementary material available at 10.1186/s12967-025-06662-5.

## Background

Breast cancer (BC) emerged as the most common cancer type diagnosed in females, in 2020 they represented 11.7% of all cancer cases [46]. In 2022, BC affected 2.3 million people globally and remained one of the leading causes of cancer death with 670,000 cases [7, 55]. The diverse and heterogeneous nature of BC pose significant challenges in predicting disease prognosis, even among patients with similar clinical subtypes [45, 56]. BC is characterized based on the prediction analysis of microarrays 50 (PAM50) using the variation of the gene expression of estrogen receptor (ER), progesterone receptor (PR), and human epidermal growth factor receptor 2 (HER2) into Luminal A, Luminal B, HER2-enriched, basal-like (triple negative), and Normal like [20, 33, 37]. Each subtype has distinct genetic alterations, clinical outcomes, and responses to therapy that impact the management of BC patients [30, 56].

In this view, modern systems biology based on omics technologies, including transcriptomics, microbiomics, and epigenomics, have accelerated the deep understanding of pathophysiological alterations in breast cancer subtypes [38]. These technologies allow the study of complex biological mechanisms related to breast cancer, identifying global biomarkers, and predict patient outcomes [40, 41].

Based on the molecular characterizations of BC, using different omics can provide a deep understanding of BC heterogeneity. Relying on a single omics dataset provides only a partial view of the disease’s progression and does not capture the latent relationships across different biological levels [61]. Thus, integrating multi-omics data is crucial for a more comprehensive understanding of BC and its subtypes [32, 44].

Despite the availability of various multi-omics integration methods, differences in performance across these approaches necessitate a detailed evaluation to identify the most effective strategy for BC subtype classification [38]. In the present study, we hypothesize  that evaluating two different approaches to multi-omics integration from the feature selection perspective and BC subtypes. The first approach is the statistical-based multi-omics factor Analysis (MOFA+) is an unsupervised multi-omics integration tool that uses latent factors to capture sources of variation across different omics modalities, offering a low-dimensional interpretation of multi-omics data [4]. The second approach is the deep learning-based multi-omics integration represented by a graph convolutional network (MoGCN) [27]. It reduces the dimensionality of the multi-omics data using an autoencoder to reduce noise and dimensionality, preserving essential features for subsequent analysis. This method emphasizes the most pertinent data points for subsequent analysis.

**Fig. 1:**
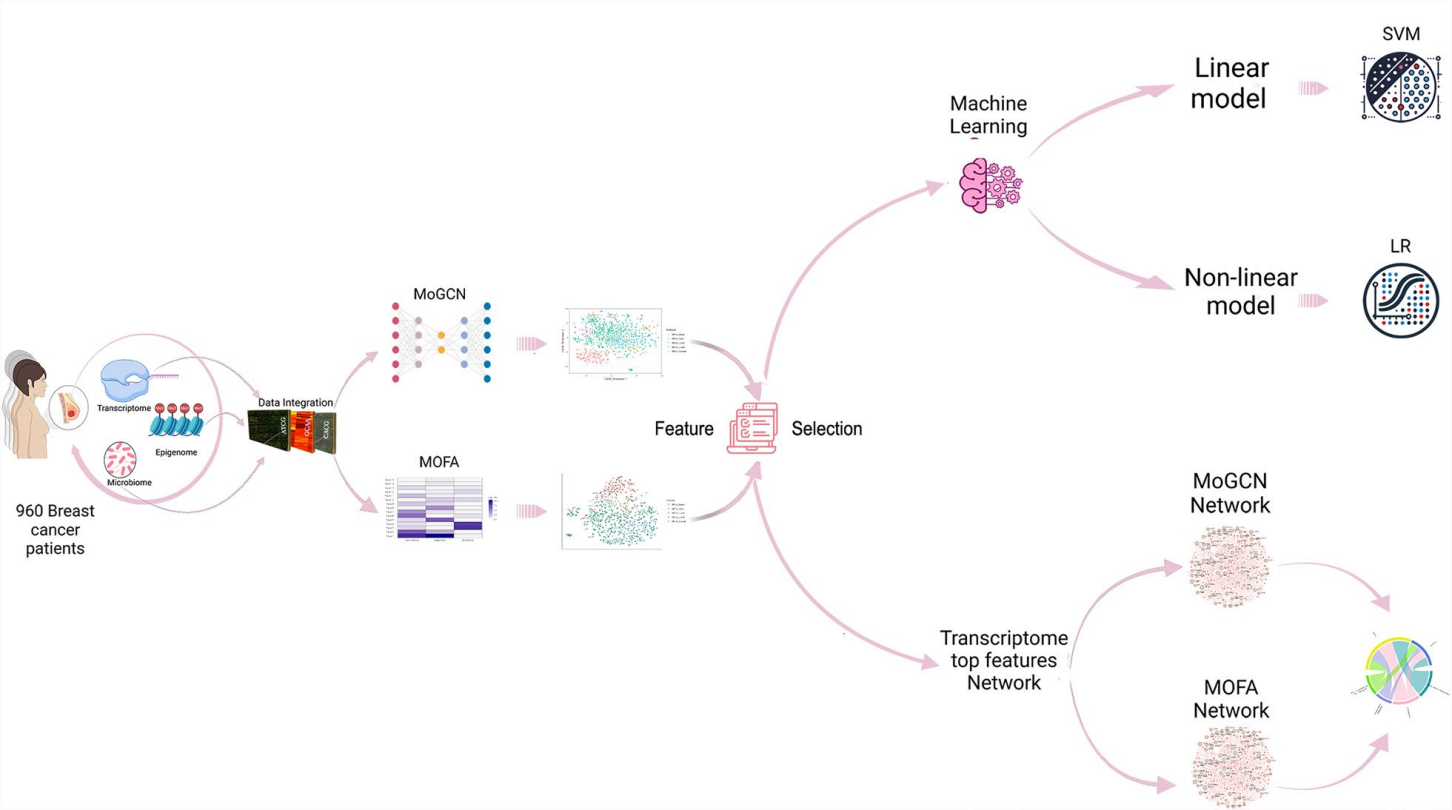
A graphical overview of the study framework. Host transcriptomics, epigenomics, and shotgun Microbiome data from 960 BC patients were obtained from TCGA through cBioPortal. These multi-omics data were integrated through two different approaches: the statistical based multi-omics Factor analysis (MOFA+) and the deep learning based multi-omics integration represented by a graph convolutional network (MoGCN). The features selected from both approaches were used to build linear (Support vector classifier (SVC) and nonlinear (Logistic Regression (LR)) machine learning models to assess the ability of the selected features to classify BC data according to subtype. Transcriptomic features from both approaches were also used to build a network analysis using OmicsNet and identify pathway enrichment related to BC subtypes

In our analysis, the features selected from both approaches were evaluated using linear and nonlinear Machine learning (ML) models. Additionally, transcriptomic features were utilized to construct networks and identify pathway enrichment related to BC subtypes (Fig. 1). Our findings suggest that integrating multi-omics data through the statistical-based approach (MOFA+) improves BC subtype classification and pathway analysis. This integration also holds promise for identifying novel biomarkers and therapeutic targets, ultimately enhancing treatment strategies for different BC subtypes.

## Methods

### Data collection

The molecular profiling including normalized host transcriptomics, epigenomics, and microbiomics data for 960 invasive breast carcinoma patient samples, sourced from The Cancer Genome Atlas (TCGA-PanCanAtlas 2018) and downloaded from the cBioPortal (https://www.cbioportal.org/) [10]. Patient samples were classified into five breast cancer with the following count per subtype: 168 Basal, 485 LumA, 196 LumB, 76 Her2, and 35 Normal_like.

### Data processing

Batch effects were corrected using unsupervised ComBat [59] through the Surrogate Variable Analysis (SVA) package (v3.50.0) [25] for both transcriptomic and microbiomics data, and Harman [39] method was implemented on methylation in order to remove the batch effect. After discarding features with zero expression in 50% of samples were discarded. After filtering, the retained features included D = 20,531 for the Transcriptome, D = 1,406 for the microbiome, and D = 22,601 for epigenome.

### Statistical-based multi-omics integration

In this approach, MOFA+ [4] is an unsupervised factor analysis method designed for the analysis of multi-omics datasets. It allows data dimensionality reduction by informative latent factors (LFs These LFs explain the variation across omics types, enabling the discovery of shared patterns and correlations across the datasets [3, 4]. We used MOFA+ package (R v 4.3.2) for unsupervised integration of the three omics datasets through driving LFs explaining data variation, allowing the extraction of feature loading score for each feature [3, 4]. LFs were selected to explain a minimum of 5% variance in at least one data type. The MOFA+ model has trained over 400,000 iterations with a convergence threshold.

### Deep learning-based integration

MoGCN integrates multi-omics data using Graph Convolutional Networks (GCNs) for cancer subtype analysis [27]. It use autoencoders for dimensionality reduction, improving feature extraction and interpretability. It calculates feature importance scores and extracts top features, merging them post-training to identify essential genes. This method can identify cancer biomarkers based on subtypes [27, 54]. In the autoencoder model, the different omics were processed using Three separate encoder-decoder pathways. Each step of the encoder or decoder is followed by a hidden layer with 100 neurons using a learning rate of 0.001.

### Feature selection for MOFA+ and MoGCN

In order to ensure a fair and consistent comparison across models, we standardize the number of selected features by extracting the top 100 features per omics layer (transcriptomics, miRNA, and methylation), resulting in a unified input of 300 features per sample for both models.

In MOFA+ [4], we selected features based on the absolute loadings from the latent factor explaining the highest shared variance across all omics layers,specifically, we used Factor one in our dataset. This approach identifies the most representative and interpretable multi-omics signals relevant to the subtyping task.

In MoGCN, we applied the built-in autoencoder based feature extractor from the MoGCN method [27] selecting the top 100 features per omics layer based on an importance score. This score was computed by multiplying the absolute encoder weights by the standard deviation of each input feature, prioritizing features with both high influence on model learning and substantial biological variability.

### Unsupervised embedding-based evaluation

To evaluate the clustering of each model, t-SNE was used alongside the Calinski-Harabasz index (Chi) [8, 43], to measure the ratio of the sum of between-cluster dispersion and within-cluster dispersion, where a higher Calinski-Harabasz score indicates better clustering performance. Additionally, the Davies-Bouldin index (DBI) [13], assesses the average similarity ratio of each cluster with its most similar cluster, with a lower Davies-Bouldin score representing better clustering quality.

### Model evaluation

The selected features derived from each approach were evaluated through two main assessment criteria. The first criterion utilizes the F1 score matrix to evaluate the performance of both linear and non-linear models. The second criterion focuses on the representation of selected features across different omics. To investigate this criterion, we utilized the Support Vector Classifier (SVC) [47] linear kernel model and Logistic regression (LR) [24], in a supervised manner to recognize complex patterns within multi-omics data, enabling them to predict the corresponding BC subtype.

#### Linear model regularization

The SVC model was trained on features generated by each method. We then used the grid search technique with the best regularization parameter for the squared L2 penalty of balanced weighted samples with 10,000 maximum iterations and a linear kernel. The SVC model was implemented using the SVC Scikit-learn package in Python 3.11.5 [42]. Grid search with fivefold cross-validation was performed, and the F1 score was used as the evaluation metric due to the unbalanced labels across BC subtypes.

#### Nonlinear model regularization

The Logistic Regression (LR) models were trained on the features generated by each method, and the grid search technique was used to search for the best regularizationstrength parameters of balanced weighted samples. The LR model was built using the LR Scikit-learn package implemented in Python 3.11.5 [42]. A grid search with fivefold cross-validation was conducted, using the F1 score as the evaluation metric to account for the imbalance in labels across BC subtypes.

### Clinical association analysis

To assess the clinical relevance of the transcriptomic features selected by MOFA+ and MoGCN, we performed a correlation and survival analysis using OncoDB [48], a curated database that links gene expression profiles to clinical features across multiple cancer types. For each model, we evaluated the top 100 transcriptomic features identified during feature selection. We tested for associations between gene expression and key clinical variables, including pathological tumor stage, lymph node involvement, metastasis stage, patient age, and race. Significance was evaluating using false discovery rate (FDR)corrected *P-values*, with a threshold of FDR < 0.05. Genes meeting this threshold were considered clinically relevant.

### Network analysis

OmicsNet 2.0 is used to construct a network interlinking the most significant features identified by a statistical-based and deep learning-based multi-omics integration approach. The IntAct database [36] enabled pathway enrichment analysis (*P-value* < 0.05) to be conducted for the respective model features, providing insights into the biological significance of the selected biomarkers [60].

## Results

### Statistical-based and deep learning-based approaches

**Fig. 2:**
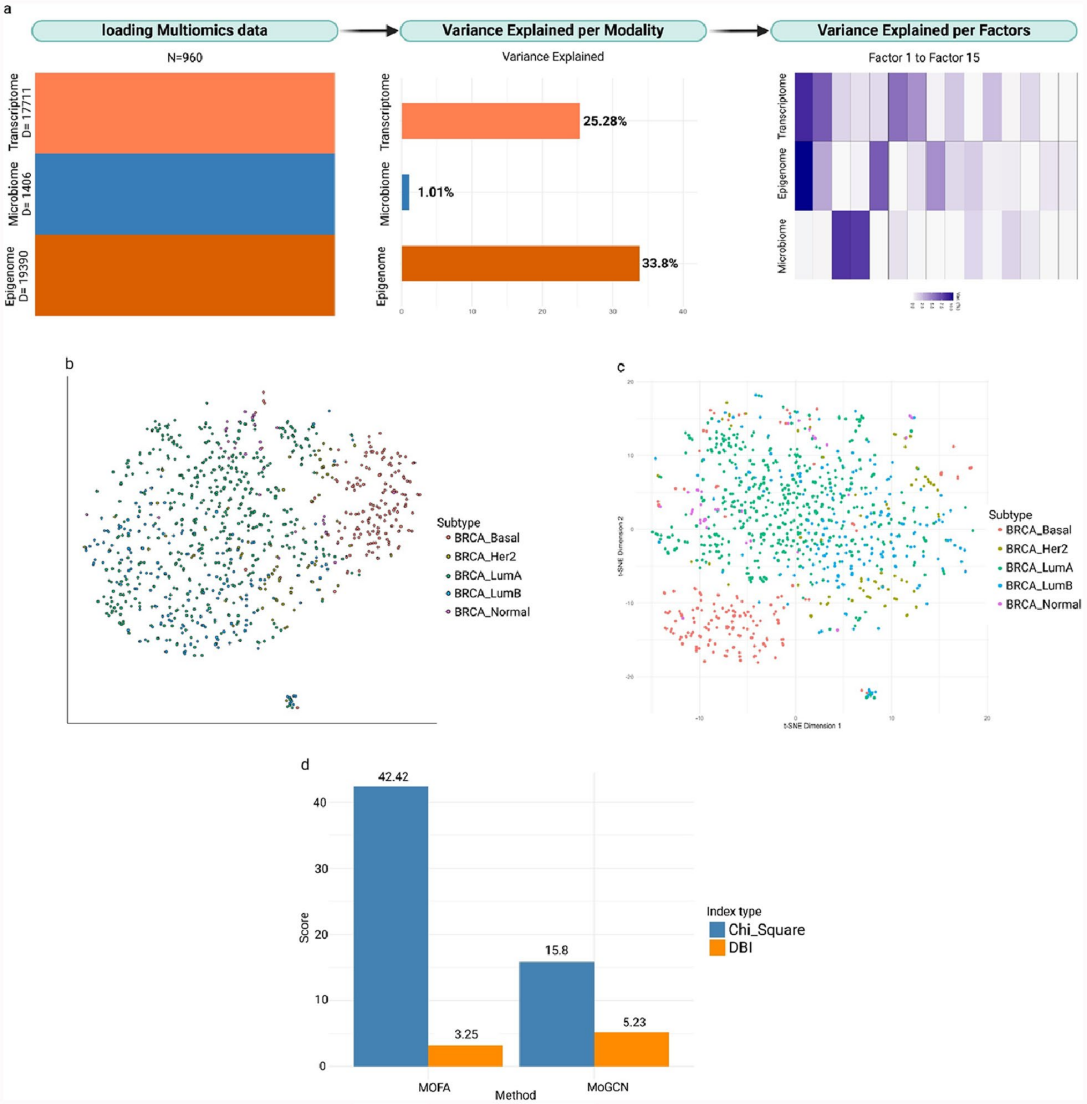
MOFA+ and MoGCN analysis of BC data*.* **a** This illustration outlines the sequential steps of the MOFA+ analysis. Starting with multi-omics data loading, the MOFA+ reduce BC multi-omics into 15 latent factors. During this process, the contribution of each factor to variance explanation is evaluated. The layers of the multi-omics dataset and a summary are shown on the left, followed by the total variance explained by each modality in the middle, and the proportion of variance explained by individual factors on the right. **b** tSNE plot illustrates the ability of MOFA+ model to classify BC data according to subtype. **c** tSNE plot illustrates the ability of MoGCN model to classify BC data according to subtype. **d** The bar plot represents the clustering ability of each model, as measured by the Chi and the DBI. The MOFA+ model achieved a higher Chi of 42.42 compared to 15.80 for MoGCN, indicating better-defined clusters. Conversely, the DBI was slightly lower for MOFA+ (3.25) than for MoGCN (3.25), suggesting marginally better cluster separation in MoGCN

MOFA+ model with 15 latent factors provided a balance between the average variance inflation factor (VIF) and the total variance explained by the MOFA+ model (Fig. 2a). Factor one explained most of BC heterogeneity (22.3 and 11.4% respectively) which captures higher variance through epigenomics than transcriptomics, followed by microbiomics (Fig. 2a). Further assessment was achieved through Distributed Stochastic Neighbor Embedding (t-SNE) visualization, which explained the clustering patterns of the data by cancer subtypes (Fig. 2b). Additionally, we extracted the autoencoder embeddings from the MoGCN model to visualize the t-SNE clustering (Fig. 2c). The MOFA+ model demonstrated a higher Chi with a value of 42.42, compared to 15.80 for the MoGCN model. The DBI was approximately the same for both MOFA+ and MoGCN, with values of 3.25 and 5.23, respectively (Fig. 2d). Further details on the features selected by MOFA+ and MOGCN in supplementary Table S1 and S2.

### Linear model classification

The results of the linear separation analysis using SVC indicate that the selected features by both MOFA+ and MoGCN cannot linearly separate between BC subtypes. The average F1 score from five-fold cross-validation for each approach is represented in Table 1. The cross-validation F1-scores for each integrator using all selected feature sets were compared against each other using t-test (*P-values* = 0.1), revealing no significant differences between them.

**Table 1:** F1 scores of SVC and LR models using all features selected from MOFA+ and MoGCN

	MOFA+	MOGCN
SVC	F1 score	F1 score
	0.556338	0.573314
LR	0.752731	0.706273

### Nonlinear model classification

**Fig. 3:**
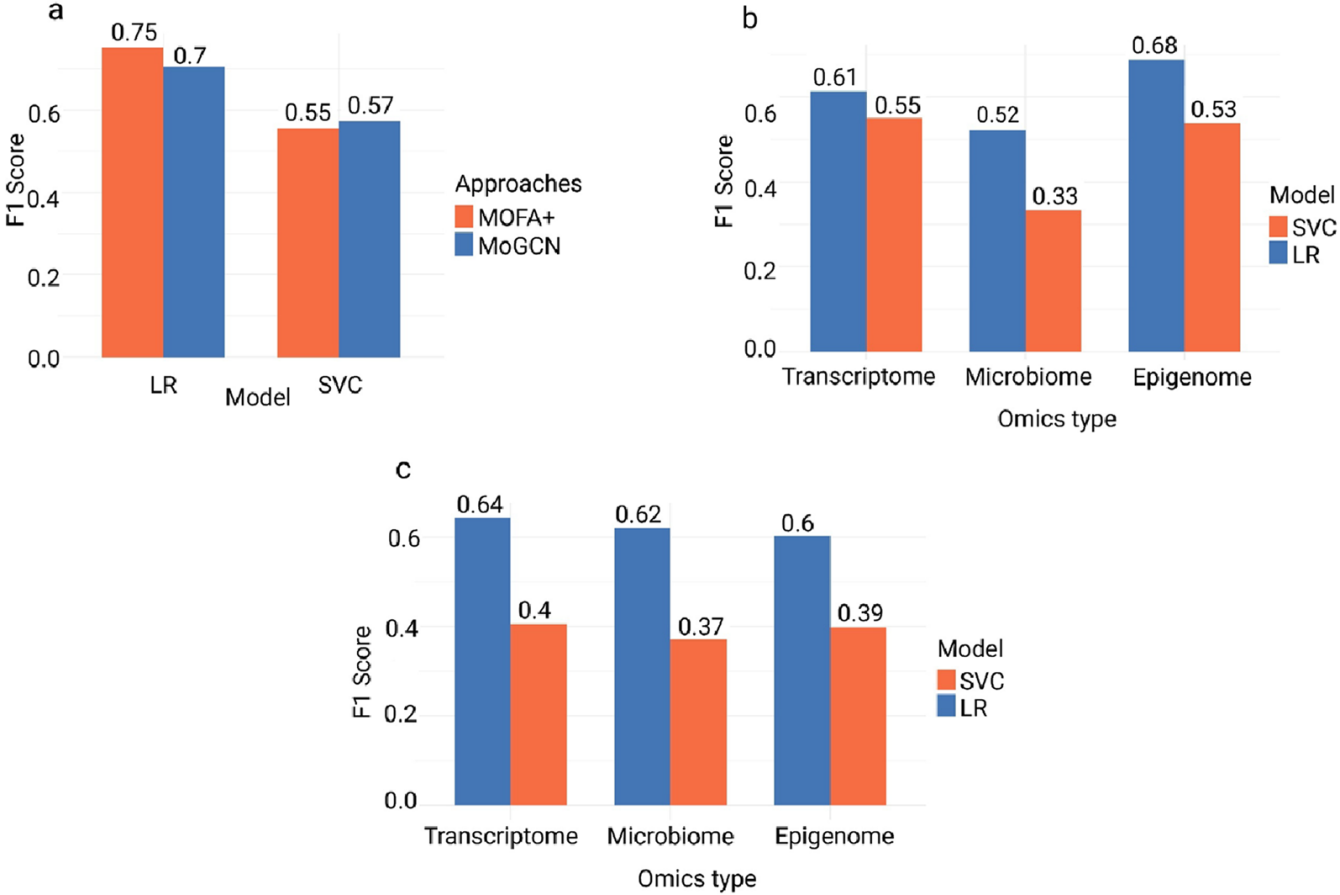
Machine learning models assessment. **a** The bar plot illustrates the F1 score for the SVC and LR for the combined selected features by features selected by the statistical-based (MOFA+) and deep learning-based (MoGCN) approaches. **b** The F1 scores for the individual omics features selected by MOFA+ are shown for both the linear model SVC and non-linear model LR, used in the classification of breast cancer data according to subtypes. **c** illustrate the F1 score for the MoGCN selected features by the individual omics also

The nonlinear separation analysis, LR shows a better performance in the classification of BC data according to subtype as indicated by a t-test (*P-value* = 0.04). LR average F1 score fivefold cross-validation is presented in Table 1 (Fig. 3a).

### Quantifying the contribution of individual omics data

In the classification task, we further quantify the contribution of individual omics. SVC failed to achieve significant classification utilizing the features selected by the individual omics across both approaches (Fig. 3b). However, LR showed better performance compared with SVC with the same features. Significant difference among different omics were observed except in the transcriptome features where there is no significant difference between the two classifiers. The *P-values* were calculated using the t-test and the average F1 score from fivefold cross-validation and test results are shown in Table 2 (Fig. 3c). Further details on the performance of other models are available in supplementary Table S3.

**Table 2:** The F1 score for qualifying for the contribution of each omics type through SVC and LR

Omics/Model Name	MOFA	MoGCN	*P-values*
SVC Transcriptome	0.5507524	0.4058878	2.37745E−05
SVC Epigenome	0.5386348	0.3973884	4.76108E−05
SVC Microbiome	0.3333358	0.370442	0.000223954
LR Transcriptome	0.6137594	0.6437164	0.054259678
LR Epigenome	0.6882982	0.60296	0.002526436
LR Microbiome	0.5226684	0.6210466	0.000414284

### Clinical associations of selected transcriptomic features

Our analysis using OncoDB demonstrated that a majority of the top transcriptomic features selected by MOFA+ (59%) and MoGCN (47%) are significantly associated with key clinical variables in breast cancer, such as tumor stage and patient demographics. These findings support the translational potential of our multi-omics models and highlight their ability to capture biologically and clinically meaningful signals that extend beyond traditional transcriptome-based classifiers. All gene-level associations and their corresponding *P-values* are provided in Supplementary Table S1.

### Network analysis

**Fig. 4:**
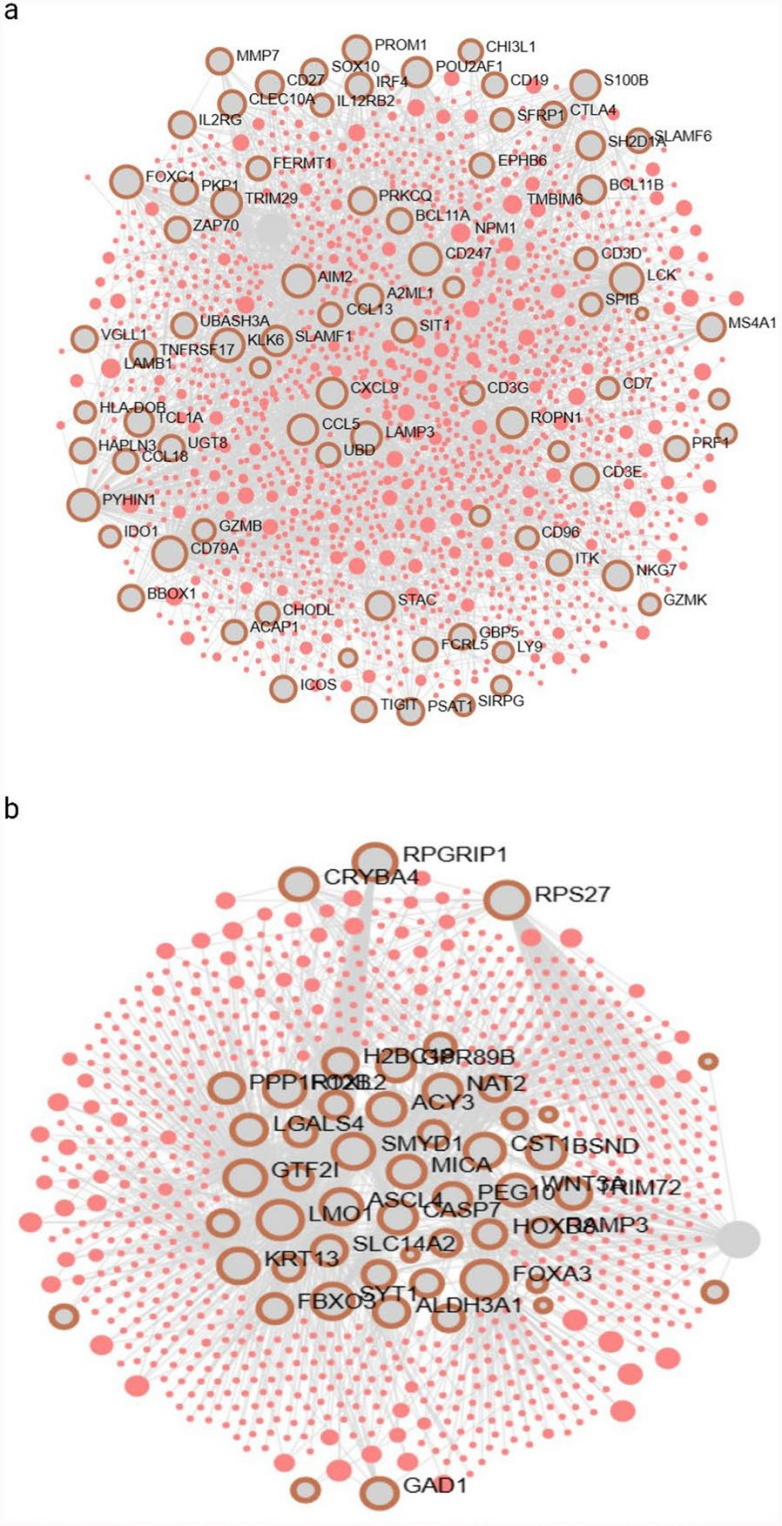
The statistical-based and deep learning-based transcriptome features selected network analysis. **a** The network shows the gene-to-protein interaction across MOFA transcriptome selected features. The network contains 1578 nodes, 2255 edges, and 90 seeds. **b** The network of MoGCN transcriptome features shows also gene to protein interactions, where the network contains 870 nodes, 1087 edges, and 60 seeds. In both networks the gray color represents genes, and the pink color represents proteins

The top 100 RNA-seq features derived from the statistical-based and deep learning-based multi-omics integration approaches were used to construct a network. The statistical based network analysis resulted in 1578 nodes, 2255 edges, and 90 seeds, and identified 121 pathways with *P-values* < 0.05 (Fig. 4a). However, the deep learning-based features constructed a network with 870 nodes, 1087 edges, and 60 seeds, revealing 100 pathways (Fig. 4b).

### Comparative network and pathway analysis among statistical-based and deep learning-based multi-omics integration approaches

**Fig. 5:**
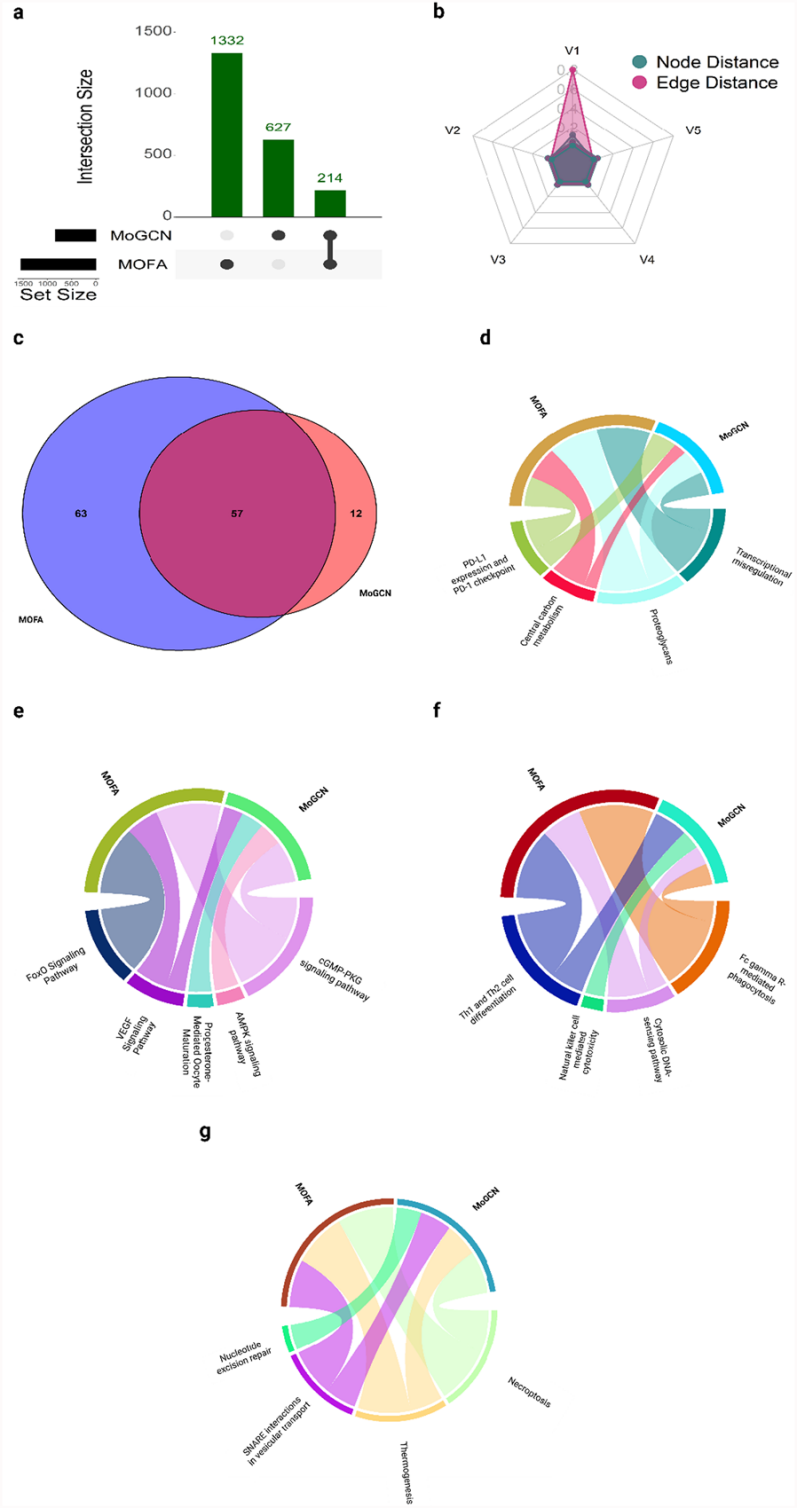
Network comparative analysis and pathway tracking analysis. **a** Upset plot comparing the node size of each network from different approaches. The statistical-based approach has the largest node size 1332 with 214 overlapping nodes between the two networks. **b** Radar plot shows the similarity between the networks on both node and edge levels based on the distances between them, the node distance is highlighted in green and the edge distance is highlighted in Pink. **c** Significant pathways (FDR < 0.05) uncovered by each method were compared to each other and represented by the Venn diagram. **d**–**g** Four pathway categories were further tracked for a better understanding of how far each method can see inside the pathway, including **d** Cancer-related Pathways, **e** Signal Transduction Pathways, **f** Immune System and Inflammation Pathways, and **g** Cellular Processes and Metabolism

The UpSet plot (Fig. 5a) showed that the statistical-based feature network has the largest node size 1332 and revealed 214 overlapping features in the intersection between the statistical-based and deep learning-based feature networks. The statistical-based feature network demonstrated a higher similarity to the deep learning-based feature network on the radar plot, both at the node and edge levels (Fig. 5b). The Venn diagram (Fig. 5c) indicated that the statistical-based and deep learning-based feature networks share 57 common significant pathways. This suggests that although the two models identified different features, they converged on common pathways, thereby highlighting more genes related to breast cancer. The pathways were categorized into four main groups: Cancer-related Pathways (Fig. 5d), Signal Transduction Pathways (Fig. 5e), Immune System and Inflammation Pathways (Fig. 5f), and Cellular Processes and Metabolism Pathways (Fig. 5g). This extensive network and its enriched pathways highlight the complex biological interactions and processes involved in breast cancer. The details of the pathways enriched in each category with the hits and FDR value are listed in Table 3. Further details on the pathways enriched from both network supplementary Table S4.

**Table 3:** The pathways enriched from the network of the statistical-based and deep learning-based transcriptome selected features with the hits and FDR values of each pathway

Pathway name	MOFA		MoGCN	
	Hits	FDR	Hits	FDR
Breast Cancer-Related Pathways				
Transcriptional Misregulation in Cancer	45	1.28E−07	22	0.00238
Proteoglycans in Cancer	43	2.51E−05	23	0.00534
Central Carbon Metabolism in Cancer	30	3.02E−08	11	0.0372
PD-L1 expression and PD-1 checkpoint pathway in cancer	28	2.01E−03	21	0.000337
Signal Transduction Pathways				
cGMP-PKG signaling pathway	54	3.46E−05	36	6.30E−05
AMPK signaling pathway	NA	NA	20	1.23E−01
Progesterone-mediated oocyte maturation	NA	NA	19	5.83E−02
VEGF signaling pathway	28	6.67E−03	16	3.94E−02
FoxO signaling pathway	59	8.35E−07	NA	NA
Immune System and Inflammation Pathways				
Fc gamma R-mediated phagocytosis	31	7.98E−09	9	0.157
Th1 and Th2 cell differentiation	30	5.09E−07	14	0.00744
Cytosolic DNA-sensing pathway	17	0.00475	8	0.157
Natural killer cell mediated cytotoxicity	NA	NA	8	0.109
Cellular Processes and Metabolism				
Necroptosis	34	0.00000248	25	0.00000611
Thermogenesis	32	0.00000025	20	0.000063
SNARE interactions in vesicular transport	28	0.000126	19	0.000329
Nucleotide excision repair	NA	NA	15	0.0239

## Discussion

In this study, we integrate a different data set including transcriptome, epigenome, and microbiome, to explore the efficacy of multi-omics integration. We utilize two different approaches: the statistical-based MOFA+ and the deep learning-based MoGCN to classify BC subtypes, including Her2, Basal, Normal-like, LumA, and LumB.

The MOFA+ model describes a significant fraction of the variance in the epigenome and transcriptome data, demonstrating a higher ability to capture the heterogeneity of BC subtypes [2, 4, 45]. The statistical approach can generate more disparate clustering of BC subtypes compared with the MoGCN model, as evidenced by the higher Chi and lower DBI associated with the MOFA+ model. These differences reflect that MOFA+ effectively balances variance inflation and total variance explained across multiple omics layers [4, 21, 52].

The complexity of the deep learning models can leads to overfitting or challenges in capturing specific subtype patterns when facing high dimensional data [15, 58]. MOGCN may lack interpretability, as it may not link latent features with biological data insights due to the fixed number of convolution filters, and the identified patterns are often correlated and redundant [1, 5]. However, MoGCN can still integrate the multi-omics data and provide valuable insights through the deep features selected, which may capture significant pathways related to BC subtypes that statistical models could overlook.

The five subtypes imbalance is one of the main limitations that we encountered which may lead to biased model performance [17]. We utilize the F1 score, a powerful metric for imbalanced datasets, as it considers both precision and recall, providing a balanced view of the model’s performance [12].

SVC as a linear model failed to capture any linear relationship for the features selected by MOFA+ and MoGCN. These may be due to the complexity of BC heterogeneity, there are no linear relationships between the selected features [6, 19].

LR performance in the classification of BC subtypes highlights the importance of the nonlinear ML model. The higher F1 score indicates that there is a nonlinear relationship among the selected features by both approaches: the statistical-based MOFA+ and deep learning-based MoGCN [19, 23, 53]. The contribution of the individual omics in the classification task emphasizes the heterogeneity of BC subtypes. SVC also failed Due to non-linear relationships on the individual omics-selected features [47]. LR as a nonlinear ML model shows that these features carry meaningful information which indicates that there is a nonlinear relationship on the individual omics level [23, 53].

Recent studies have shown that PAM50 subtypes, particularly HER2-enriched and Luminal B, often exhibit high intra-group heterogeneity, limiting their clinical classification accuracy [50, 51]. To assess potential improvements, we compared the predictive performance of integrated multi-omics features selected by MOFA+ and MoGCN to transcriptome-only features aligned with PAM50. Our results demonstrate that multi-omics integration offers better classification: using MOFA+, the F1 score increased by 32% for HER2 and 37.9% for Luminal B; with MoGCN, improvements of 27.6% for HER2 and 15.5% for Luminal B were observed. These findings suggest that multi-omics approaches could provide a more refined subtyping, particularly for subtypes known for variable therapeutic responses. In addition to supervised classification, clustering quality was also evaluated using direct comparison of Latent representations derived from PCA applied to transcriptome-only features achieved a CHI of 11.06, whereas MOFA+ and MoGCN models achieved higher CHI scores of 42.2 and 15.8, respectively. These results indicate that multi-omics models produce more distinct and well-separated clusters. Detailed subtype-specific performance metrics and clustering comparisons are provided in Supplementary Tables S1 and S5.

Despite promising performance metrics, applying machine learning models to clinical omics data presents important challenges that must be addressed before translation into practice. One key issue is the inconsistency in clinical annotations across public datasets such as TCGA. Heterogeneous data labels, non-standardized terminology, and missing metadata introduce noise that may limit model generalization. Previous studies have shown that the use of standardized medical ontologies such as Unified Medical Language System (UMLS) and SNOMED CT can enhance the harmonization of the data across cohorts, and improving model training and reproducibility [9].

In addition, while F1 scores are widely used to evaluate predictive models, they provide limited insight into biological or clinical validity. F1 scores do not capture the consequences of false positives or false negatives in a healthcare setting, nor do they reflect interoperability which is an essential requirement for clinical deployment [49]. In this prospective, model interpretation techniques such as Shapley additive explanation (SHAP) can enhance interpretability [35] by quantifying the contribution of each molecular feature to a prediction, thus offering supporting clinical decision making.

The OmicsNet network analysis derived from the features selected by both approaches highlights significant insights into the biological pathways associated with BC subtypes. The statistical-based network has a large node size and more complex captures a wide range of biological interactions. MOFA can provide more comprehensive insights into the heterogeneity of breast cancer subtypes [4]. In contrast, the deep learning-based network, which is more focused and smaller network, still captures key pathways related to the BC subtype. MoGCN primary focus is on minimizing reconstruction error can lead to the extraction of features that are less interpretable or biologically meaningful. This can sometimes limit the depth of integration compared to MOFA’s factor analysis approach [11, 26].

The different methodologies identified an overlapping pathway involved in cancer-related processes, signal transduction, immune responses, and cellular metabolism are retrieved from both approaches.

One of the potential pathways enriched from both approaches is The Fc gamma R-mediated phagocytosis pathway. Fc gamma receptors (FcγRs) are immune cell receptors [16]. These receptors recognize and bind to the Fc region of immunoglobulin G (IgG) antibodies, which coat target cells such as pathogens or cancer cells through a process called opsonization [18]. In breast cancer, this process can occur naturally. Once FcγRs on immune cells bind to antibody-coated cancer cells, a signaling cascade is triggered, involving immunoreceptor tyrosine-based activation motifs (ITAMs) and spleen tyrosine kinase (Syk) which enable the immune cell to engulf and degrade the cancer cell [22, 31]. According to our knowledge, it has limited information available on its role in breast cancer subtypes. However, this pathway’s involvement in immune response characteristics suggests potential opportunities for immunotherapy, particularly in HER2+ breast cancer subtypes [34].

Additionally, SNARE (Soluble NSF Attachment Protein Receptor) pathway plays a critical role in cancer progression. It regulates tumor cell migration and invasion by influencing the formation of cellular protrusions and the secretion of matrix metalloproteinases that degrade the extracellular matrix [29]. In angiogenesis, SNARE proteins facilitate the secretion of angiogenic factors like VEGF, promoting new blood vessel formation to support tumor growth [14]. They also modulate tumor cell communication through the release of exosomes, which can alter the tumor microenvironment and immune response [28, 57]. Future work should focus on validating these pathways in independent datasets and exploring their functional roles in BC progression and treatment response. Future research should focus on several key areas to advance the understanding and clinical application of multi-omics integration in breast cancer (BC). Validating the identified pathways, such as Fc gamma R-mediated phagocytosis and the SNARE pathway, in independent datasets is crucial for confirming their relevance and functional roles in BC subtype. Enhancing multi-omics integration through novel algorithms and improving model interpretability with advanced techniques will address current limitations.

## Conclusion

In this study, we successfully integrated multi-omics data, including transcriptome, epigenome, and microbiome profiles, to investigate breast cancer (BC) subtypes using both statistical-based and deep learning-based approaches. Our findings highlight the complementary strengths and limitations of the MOFA+ and MoGCN models in capturing the heterogeneity of BC subtypes.

The MOFA+ model demonstrated superior performance in explaining the variance within the multi-omics data and provided more robust clustering of BC subtypes, as evidenced by its higher Chi and balanced variance inflation factors. Conversely, the MoGCN model, while capturing significant features through deep learning, faced challenges related to interpretability and potential overfitting due to its complexity.

Linear models, such as SVC, were inadequate for capturing the complex relationships between features, underscoring the need for nonlinear approaches. The Logistic Regression (LR) model’s superior performance emphasizes the importance of considering nonlinear interactions in classifying BC subtypes. Additionally, our analysis of individual omics contributions revealed that LR effectively leverages meaningful information from diverse omics layers, further supporting the value of nonlinear models in this context.

The network and pathway analysis provided valuable insights into the biological mechanisms underlying BC heterogeneity. Notably, pathways such as Fc gamma R-mediated phagocytosis and SNARE interactions were identified as significant across both statistical and deep learning approaches. These pathways not only highlight critical immune and cellular processes but also offer potential targets for therapeutic interventions and biomarker discovery.

Overall, this study underscores the importance of integrating multi-omics data and employing a combination of statistical and machine learning approaches to unravel the complexity of breast cancer. Future research should focus on validating these findings in independent datasets and exploring the functional roles of identified pathways to advance our understanding of BC and improve personalized treatment strategies.

## Supplementary Information

Additional file 1.

## Data Availability

The datasets analysed during the current study are available in the Cancer Genome Atlas (TCGA-PanCanAtlas 2018) and downloaded from the cBioPortal (https://www.cbioportal.org/). The codes used are available through get-hub (https://github.com/mahmoudmohsen33/Comparative-Analysis-of-Statistical-and-Deep-Learning-Based-Multi-Omics-Integration-for-Breast-Cance).

## Abbreviations

BC: Breast Cancer
Chi: Calinski-Harabasz Index
DBI: Davies-Bouldin Index
ER: Estrogen Receptor
FcγRs: Fc gamma receptors
FDR: False Discovery Rate
GCN: Graph Convolutional Network
HER2: Human Epidermal Growth Factor Receptor 2
IgG: Immunoglobulin G
ITAM: Immunoreceptor Tyrosine-based Activation Motif
LR: Logistic Regression
ML: Machine Learning
MOFA+: Multi-Omics Factor Analysis Plus
MoGCN: Multi-Omics Graph Convolutional Network
NSF: N-Ethylmaleimide-Sensitive Factor
*P-value*: Probability Value
PAM50: Prediction Analysis of Microarrays 50
PR: Progesterone Receptor
RF: Random Forest
SNARE: Soluble NSF Attachment Protein Receptor
SVC: Support Vector Classifier
Syk: Spleen Tyrosine Kinase
t-SNE: T-Distributed Stochastic Neighbor Embedding
VEGF: Vascular Endothelial Growth Factor
SHAP: Shapley additive explanation
UMLS: Unified Medical Language System
