# Supplementary material for: Comparative analysis of statistical and deep learning-based multi-omics integration for breast cancer subtype classification
Source: J Transl Med. 2025 Jul 1;23:709. doi: 10.1186/s12967-025-06662-5 (PMC12210783; doi:10.1186/s12967-025-06662-5)
Supplement: Supplementary file 1 — Additional file 1. [file 12967_2025_6662_MOESM1_ESM.docx]

Comparative Analysis of Statistical and Deep Learning-Based Multi-Omics Integration for Breast Cancer Subtype Classification

Mahmoud M. Omran^1,2,3^, Mohamed Emam^1,4,5^, Mariam Gamaleldin^2^, Asmaa M. Abushady^2^, Mustafa A. Elattar^3,6^, Mohamed El-Hadidi ^1,7^

^1^ Bioinformatics group, Center for Informatics Sciences (CIS), Nile University, Giza, Egypt

^2^ School of Biotechnology, Nile University, Giza, Egypt

^3^School of Information Technology and Computer Science, Nile University, Giza, Egypt

^4^ Current Adress CIIMAR/CIMAR, Interdisciplinary Centre of Marine and Environmental Research, University of Porto, Terminal de Cruzeiros do Porto de Leixões, Av. General Norton de Matos, s/n, 4450-208 Porto, Portugal

^5^ Current Adress Department of Biology, Faculty of Sciences, University of Porto, Rua do Campo Alegre, 4169-007 Porto, Portugal

^6^Medical Imaging and Image Processing Research Group, Center for Informatics Science, Nile University, Giza, Egypt

^7^ Current Institute of Cancer and Genomic Sciences, College of Medical and Dental Sciences, University of Birmingham Dubai Campus, Dubai, United Arab Emirates.

**Supplementary Tables**

**Supplementary Table S1.** Clinical associations of transcriptomic features selected by MOFA+ and MoGCN models. The table lists a subset of genes from the top 100 transcriptomic features identified by each model. “Status” indicates whether the gene was found to have a statistically significant association (ANOVA p-value < 0.05) with any clinical parameter based on external clinical datasets. Associated clinical parameters (e.g., age, race, pathological stage, TNM stages) are listed alongside their corresponding ANOVA p-values. These associations were cross-referenced with OncoDB to assess the biological and clinical relevance of the selected features.

| MOFA | | | | MoGCN | | | |
| --- | --- | --- | --- | --- | --- | --- | --- |
| Gene | Status | Clinical Parameters | ANOVA_pvalues | Gene | Status | Clinical Parameters | ANOVA pvalues |
| BCL11A | YES | Age; Race | 9.3e-05; 7.6e-04 | C3orf72 | NO |  |  |
| GABRP | YES | Age; Race | 4.9e-02; 1.1e-04 | AMAC1L3 | NO |  |  |
| VGLL1 | NO |  |  | BSND | YES | Pathological M stage; Pathological N stage; Pathological stage; Pathological T stage | 1.1e-08; p<=1.0e-20; 7.1e-06; p<=1.0e-20 |
| GZMB | YES | Pathological M stage; Pathological stage | 2.6e-02; 3.3e-03 | FOXL2 | NO |  |  |
| PSAT1 | YES | Age; Race | 2.9e-03; 7.3e-03 | ID2B | NO |  |  |
| SFRP1 | YES | Age; Race | 4.1e-03; 4.8e-02 | SFTPB | YES | Pathological N stage | 2.30E-02 |
| CD38 | YES | Pathological M stage; Pathological T stage; Race | 1.5e-05; 3.1e-03; 1.6e-03 | WNT3A | NO |  |  |
| FCRL5 | YES | Pathological M stage; Race | 2.9e-11; 7.1e-04 | PPIAL4C | YES | Pathological stage; Race | 1.3e-02; 1.4e-02 |
| PLA2G2D | NO |  |  | KRT13 | NO |  |  |
| LAMP3 | YES | Age; Race | 5.9e-08; 1.1e-02 | OR10AD1 | NO |  |  |
| POU2AF1 | YES | Pathological M stage; Race | 4.0e-10; 1.8e-03 | RGAG1 | NO |  |  |
| PPP1R14C | YES | Age; Race | 5.1e-03; 6.8e-05 | DCAF4L1 | NO |  |  |
| IL12RB2 | YES | Pathological N stage; Pathological stage | 3.5e-02; 4.4e-02 | ALDH3A1 | NO |  |  |
| ROPN1 | YES | Race | 3.50E-04 | C22orf24 | NO |  |  |
| IDO1 | NO |  |  | ANO3 | YES | Race | 9.30E-03 |
| SPIB | YES | Pathological T stage | 4.30E-02 | CRYBA4 | YES | Pathological M stage; Pathological N stage; Pathological stage; Pathological T stage; Race | 9.0e-08; p<=1.0e-20; 7.4e-04; p<=1.0e-20; 5.6e-03 |
| CXCL9 | YES | Pathological M stage | 3.20E-02 | GOLGA9P | NO |  |  |
| CD79A | YES | Pathological M stage; Race | 5.7e-03; 1.9e-03 | ZSCAN10 | YES | Pathological T stage | 4.50E-04 |
| RARRES1 | YES | Pathological M stage | 8.40E-03 | ABCB11 | YES | Pathological N stage; Pathological stage | 3.0e-02; 3.2e-02 |
| IRF4 | YES | Pathological T stage | 3.10E-04 | MICA | YES | Pathological N stage | 2.20E-02 |
| PROM1 | YES | Age | 1.20E-03 | PGAM4 | NO |  |  |
| BMS1P20 | NO |  |  | DDX11L2 | NO |  |  |
| HLA-DOB | YES | Race | 6.80E-03 | SLC14A2 | NO |  |  |
| SOX10 | YES | Age; Pathological N stage; Race | 3.4e-03; 4.9e-02; 1.0e-02 | NBPF6 | YES | Pathological N stage | 2.10E-02 |
| MARCO | YES | Race | 1.20E-02 | FKBP1AP1 | NO |  |  |
| BBOX1 | YES | Pathological T stage | 5.40E-06 | ARSJ | NO |  |  |
| GBP5 | NO |  |  | FBXL22 | YES | Age; Pathological T stage; Race | 3.9e-04; 1.9e-02; 3.5e-02 |
| CD19 | YES | Race | 1.60E-02 | C2orf43 | NO |  |  |
| MS4A1 | NO |  |  | PROZ | YES | Pathological M stage | 3.70E-03 |
| A2ML1 | NO |  |  | NBPF16 | NO |  |  |
| HAPLN3 | YES | Race | 5.20E-06 | CASP7 | YES | Pathological M stage; Pathological T stage | 1.3e-02; 2.2e-02 |
| TIGIT | NO |  |  | C1orf116 | YES | Gender | 8.40E-06 |
| ADAMDEC1 | YES | Race | 3.60E-02 | C3orf48 | NO |  |  |
| PRKCQ | YES | Gender | 5.00E-02 | TUBA3C | YES | Pathological M stage | 6.30E-03 |
| UBD | YES | Pathological N stage | 4.90E-02 | FOXA3 | YES | Pathological N stage | 3.50E-03 |
| FOXC1 | YES | Age; Race | 4.8e-02; 3.6e-06 | CITED1 | NO |  |  |
| TRIM29 | YES | Age; Race | 3.3e-02; 2.5e-03 | LOC644936 | NO |  |  |
| AIM2 | NO |  |  | ANP32AP1 | NO |  |  |
| FCRL3 | NO |  |  | WFDC10B | YES | Age | 2.80E-12 |
| CCL19 | YES | Age | 1.60E-02 | IFNB1 | YES | Age; Pathological N stage; Pathological T stage | 2.9e-02; 3.6e-02; 3.5e-06 |
| CHI3L1 | NO |  |  | BCO2 | NO |  |  |
| FCRLA | NO |  |  | KLHDC7A | YES | Gender | 1.50E-04 |
| SIRPG | NO |  |  | ARHGAP36 | YES | Age; Pathological stage | 1.6e-03; 1.9e-02 |
| CCL18 | NO |  |  | ZFP57 | YES | Pathological M stage; Pathological N stage; Pathological stage; Pathological T stage; Race | 4.5e-06; p<=1.0e-20; 8.0e-04; p<=1.0e-20; 2.1e-02 |
| MMP7 | YES | Pathological T stage; Race | 7.5e-09; 2.9e-06 | CHRNE | YES | Race | 2.10E-02 |
| SLAMF1 | NO |  |  | GPR89B | NO |  |  |
| SLAMF6 | NO |  |  | SMYD1 | YES | Age; Pathological T stage | 9.8e-03; 1.4e-08 |
| TNFRSF17 | YES | Pathological M stage; Pathological T stage; Race | 3.4e-03; 3.5e-02; 5.5e-05 | TBC1D3P1 | NO |  |  |
| LCK | NO |  |  | PPP1R12B | YES | Race | 3.90E-06 |
| SH2D1A | NO |  |  | PRR16 | NO |  |  |
| ICOS | NO |  |  | CST1 | YES | Race | 5.80E-09 |
| KIAA0125 | NO |  |  | FBXO3 | YES | Pathological M stage; Race | 4.9e-02; 1.7e-06 |
| CCL13 | YES | Pathological T stage | 4.70E-06 | GCM1 | NO |  |  |
| RASAL1 | YES | Age; Pathological stage; Pathological T stage; Race | 5.1e-04; 7.5e-03; 2.5e-02; 5.2e-04 | CHAD | YES | Age | 2.70E-02 |
| CD3E | NO |  |  | PTPLAD2 | NO |  |  |
| STAC | YES | Pathological N stage | 1.20E-02 | HSPC157 | NO |  |  |
| PLAC8 | YES | Age; Pathological stage | 2.0e-03; 4.1e-02 | SYT1 | NO |  |  |
| ROPN1B | YES | Race | 4.10E-03 | C17orf67 | YES | Gender; Pathological M stage; Pathological N stage; Pathological stage; Pathological T stage | 1.0e-04; 1.1e-04; 2.2e-08; 1.8e-03; 4.6e-09 |
| SLC34A2 | YES | Age | 1.20E-02 | GPR44 | NO |  |  |
| ITK | NO |  |  | SPDYE7P | NO |  |  |
| UBASH3A | NO |  |  | ASCL4 | NO |  |  |
| TCL1A | NO |  |  | HERC2P4 | NO |  |  |
| PYHIN1 | NO |  |  | GRIK5 | YES | Pathological T stage | 4.20E-02 |
| CD79B | YES | Pathological M stage; Race | 4.8e-02; 4.2e-05 | RPS27 | YES | Gender; Pathological N stage | 4.4e-02; 3.2e-02 |
| ZAP70 | YES | Race | 8.40E-05 | ADAM20 | YES | Pathological M stage; Pathological stage | 3.3e-02; 4.7e-02 |
| UGT8 | YES | Age; Pathological N stage; Pathological stage; Pathological T stage | 1.0e-02; 5.2e-03; 4.8e-04; 4.4e-03 | GCNT7 | YES | Pathological stage | 1.20E-02 |
| S100B | NO |  |  | HOXD8 | YES | Race | 3.10E-04 |
| CD3G | NO |  |  | LOC284009 | NO |  |  |
| ACAP1 | YES | Pathological M stage; Race | 1.9e-02; 1.1e-04 | GRK4 | YES | Pathological T stage; Race | 4.0e-02; 2.3e-02 |
| BCL11B | YES | Age; Gender | 4.9e-02; 5.0e-02 | GAD1 | NO |  |  |
| CD96 | NO |  |  | ADAM29 | NO |  |  |
| PRF1 | NO |  |  | C1orf223 | NO |  |  |
| LY9 | NO |  |  | PDZRN4 | YES | Pathological N stage | 2.40E-02 |
| CHODL | YES | Race | 2.90E-04 | NAT2 | NO |  |  |
| CD27 | YES | Pathological M stage; Race | 3.1e-02; 2.4e-02 | RASL12 | YES | Age | 2.20E-02 |
| IL2RA | YES | Race | 7.20E-03 | RAMP3 | NO |  |  |
| CD3D | NO |  |  | ARPP21 | NO |  |  |
| BTLA | NO |  |  | FAM157A | NO |  |  |
| EPHB6 | YES | Race | 3.10E-02 | OR13A1 | YES | Pathological N stage; Pathological stage | 1.3e-04; 7.8e-11 |
| CLEC10A | YES | Age; Pathological stage; Pathological T stage | 1.8e-03; 1.6e-03; 1.9e-02 | HIST2H2BF | NO |  |  |
| NKG7 | YES | Race | 2.10E-02 | ACY3 | YES | Pathological N stage | 8.90E-03 |
| FERMT1 | YES | Race | 6.10E-04 | RLN1 | NO |  |  |
| CCL5 | NO |  |  | C4orf19 | YES | Age; Pathological M stage | 1.7e-02; 3.0e-02 |
| IL22RA2 | NO |  |  | ABCC13 | NO |  |  |
| TRAT1 | NO |  |  | GTF2I | YES | Pathological M stage; Race | 4.9e-04; 7.1e-11 |
| SPOCK2 | YES | Pathological stage | 4.30E-02 | GOLGA2P6 | NO |  |  |
| ELF5 | YES | Pathological N stage; Pathological T stage; Race | 1.3e-03; 3.5e-02; 2.2e-02 | TPSD1 | NO |  |  |
| SIT1 | NO |  |  | FAM19A1 | NO |  |  |
| EOMES | NO |  |  | SCGB1D2 | YES | Pathological N stage | 2.30E-04 |
| CTLA4 | YES | Race | 3.80E-03 | LGALS4 | YES | Pathological M stage; Pathological N stage; Race | 1.3e-06; 2.5e-05; 5.2e-10 |
| IL2RG | NO |  |  | RPGRIP1 | NO |  |  |
| PKP1 | YES | Race | 2.50E-06 | PEG10 | NO |  |  |
| ZNF831 | NO |  |  | PIWIL2 | YES | Gender; Pathological T stage | 2.6e-02; 1.6e-02 |
| ART3 | YES | Race | 4.00E-06 | C9orf153 | NO |  |  |
| CD247 | NO |  |  | CT62 | YES | Race | 9.20E-04 |
| CD7 | YES | Race | 8.60E-07 | TAL2 | YES | Race | 1.90E-02 |
| LTB | YES | Pathological stage; Race | 2.0e-11; 4.6e-05 | FGF18 | NO |  |  |
| GZMK | NO |  |  | TRIM72 | YES | Pathological N stage; Pathological stage | 1.2e-02; 9.0e-07 |
| KLK6 | YES | Pathological N stage; Pathological T stage; Race | 5.3e-04; 2.3e-02; 2.4e-03 | LMO1 | YES | Pathological T stage; Race | 8.1e-05; 2.7e-04 |
| IGF2BP2 | YES | Race | 2.10E-03 | CXCL13 | YES | Age | 2.40E-02 |

**Supplementary Table S2.** Top 100 features selected by MOFA+ and MoGCN from the microbiomics and epigenomics data layers. Features were ranked based on their absolute factor loadings in MOFA+ and encoder-based importance scores in MoGCN. These features represent the most informative variables contributing to the latent structure of each model and were used in the classification analysis.

| MOFA | MOGCN |
| --- | --- |
| Microbiome | |
| Dethiosulfatarculus | Lachnoclostridium |
| Marivirga | Aeromonas |
| Candidatus_Profftella | Chitinivibrio |
| Actinoplanes | Luteibacter |
| Synergistes | Campylobacter |
| Candidatus_Hepatobacter | Sunxiuqinia |
| Pleomorphomonas | Terrabacter |
| Prasinovirus | Microvirga |
| Natronolimnobius | Whispovirus |
| Lactobacillus | Nitrospira |
| Desulfospira | Apibacter |
| Legionella | Marichromatium |
| Virgibacillus | Wolbachia |
| Pontibacter | Ruegeria |
| Citreicella | Ornithobacterium |
| Afifella | Saccharibacter |
| Candidatus_Solibacter | Polyomavirus |
| Demequina | Candidatus_Nitrosopelagicus |
| Reyranella | Crocosphaera |
| Sediminibacterium | Histophilus |
| Saccharomonospora | Alpharetrovirus |
| Tatlockia | Succinimonas |
| Jiangella | Brucella |
| Oceanicaulis | Sorangium |
| Thioalkalivibrio | Collimonas |
| Palaeococcus | Vibrio |
| Halorhodospira | Prosthecomicrobium |
| Proboscivirus | Simplexvirus |
| Gemmobacter | Sanguibacteroides |
| Dictyoglomus | Xylella |
| Holophaga | Flectobacillus |
| Desulfocarbo | Ureaplasma |
| Oerskovia | Blautia |
| Haloarcula | Loktanella |
| Desulfomonile | Grimontia |
| Actinosynnema | Rickettsia |
| Geovibrio | Anaplasma |
| Succinimonas | Proteus |
| Leeia | Thermacetogenium |
| Hypovirus | Riemerella |
| Octadecabacter | Acetobacter |
| Klebsiella | Francisella |
| Pandoraea | Paludibacter |
| Thiorhodovibrio | Fodinicurvata |
| Turneriella | Actinobacillus |
| Paramesorhizobium | Arenibacter |
| Catellicoccus | Sodalis |
| Sutterella | Legionella |
| Porphyrobacter | Cucumovirus |
| Thalassospira | Caldanaerobacter |
| Faecalibaculum | Gordonia |
| Actibacterium | Gammaretrovirus |
| Tolumonas | Capnocytophaga |
| Kallipyga | Pyrolobus |
| Phycicoccus | Plesiocystis |
| Amycolatopsis | Coleofasciculus |
| Chthonomonas | Sulfurihydrogenibium |
| Blastomonas | Olleya |
| Haloferula | Chlamydia |
| Halioglobus | Tropicibacter |
| Orenia | Methanocella |
| Planktothricoides | Brackiella |
| Cellulomonas | Mamastrovirus |
| Aquimarina | Exiguobacterium |
| Intrasporangium | Parvimonas |
| Tomitella | Kurthia |
| Butyrivibrio | Hafnia |
| Orthopoxvirus | Phycicoccus |
| Hyphomonas | Bracovirus |
| Zymobacter | Hyphomonas |
| Desulfarculus | Colwellia |
| Achromobacter | Orthohepadnavirus |
| Promicromonospora | Gemmata |
| Alpharetrovirus | Betapartitivirus |
| Alphapapillomavirus | Sutterella |
| Methylocaldum | Acidithiobacillus |
| Aeromonas | Natronorubrum |
| Candidatus_Stoquefichus | Thermodesulfobacterium |
| Alloactinosynnema | Treponema |
| Myxococcus | Desulfuromonas |
| Nafulsella | Ignicoccus |
| Methanocella | Mastigocoleus |
| Micromonospora | Hypovirus |
| Collimonas | Pseudonocardia |
| Kamptonema | Actinomycetospora |
| Entomoplasma | Coprococcus |
| Phaseolibacter | Alphapapillomavirus |
| Alphacoronavirus | Mageeibacillus |
| Algicola | Raoultella |
| Parvimonas | Candidatus_Symbiobacter |
| Actinopolymorpha | Ranavirus |
| Lymphocryptovirus | Anaerococcus |
| Flammeovirga | Spiribacter |
| Prosthecomicrobium | Flavivirus |
| Luteibacter | Hapalosiphon |
| Marichromatium | Roseivirga |
| Tropicibacter | Lymphocryptovirus |
| Ruegeria | Elizabethkingia |
| Terrabacter | Enterococcus |
| Lachnoclostridium | Metallosphaera |
| Epigenome | |
| cg22294908 | cg23365832 |
| cg01078434 | cg08085165 |
| cg16112157 | cg05852416 |
| cg17240454 | cg08222185 |
| cg02554564 | cg06538003 |
| cg00497084 | cg00156216 |
| cg18328334 | cg00176879 |
| cg01637734 | cg00537910 |
| cg11719784 | cg09253125 |
| cg19099213 | cg14992108 |
| cg20895028 | cg02720618 |
| cg06415153 | cg22063056 |
| cg07361385 | cg26847093 |
| cg13904968 | cg09462826 |
| cg25682080 | cg01107031 |
| cg08367223 | cg05417615 |
| cg04806409 | cg25636075 |
| cg20092728 | cg17339202 |
| cg03998348 | cg12480658 |
| cg00953256 | cg19490266 |
| cg24607535 | cg00940891 |
| cg18239253 | cg18984499 |
| cg16979445 | cg17741572 |
| cg03242666 | cg24678320 |
| cg24525573 | cg23196133 |
| cg24835159 | cg06572974 |
| cg16708623 | cg02034222 |
| cg08615333 | cg06502868 |
| cg08784110 | cg15427448 |
| cg05606799 | cg03283569 |
| cg04458548 | cg12351042 |
| cg17423978 | cg20972553 |
| cg22214414 | cg17966192 |
| cg22580512 | cg16842485 |
| cg14494812 | cg08934427 |
| cg04184278 | cg11206634 |
| cg24269657 | cg05189291 |
| cg04574507 | cg17166338 |
| cg11802013 | cg22796458 |
| cg06022562 | cg18411891 |
| cg07347645 | cg19407886 |
| cg21201572 | cg14338062 |
| cg24459209 | cg21743649 |
| cg12610744 | cg22311533 |
| cg00237010 | cg07640820 |
| cg04345475 | cg14159672 |
| cg26946769 | cg05288803 |
| cg08474603 | cg19403023 |
| cg08404225 | cg03309967 |
| cg18129786 | cg07679836 |
| cg06436504 | cg22718139 |
| cg03109316 | cg15052335 |
| cg26091981 | cg18357098 |
| cg17582777 | cg08315277 |
| cg02682905 | cg21459921 |
| cg07237830 | cg10078415 |
| cg02431964 | cg26824091 |
| cg26511075 | cg14356550 |
| cg14472778 | cg10970251 |
| cg13007988 | cg17217677 |
| cg05953243 | cg10515956 |
| cg16862361 | cg03038672 |
| cg11584936 | cg12187567 |
| cg12285118 | cg08278554 |
| cg21307628 | cg11617144 |
| cg11435943 | cg02422627 |
| cg25995212 | cg15262516 |
| cg20579480 | cg19318511 |
| cg03503295 | cg26631477 |
| cg18085435 | cg23126915 |
| cg24579667 | cg04739485 |
| cg15830940 | cg04223956 |
| cg24928687 | cg04511534 |
| cg03468463 | cg23894539 |
| cg01103730 | cg17321617 |
| cg00673191 | cg18660898 |
| cg25219333 | cg15754084 |
| cg26767897 | cg24512303 |
| cg11547724 | cg17466768 |
| cg06119575 | cg25836326 |
| cg10071275 | cg23128056 |
| cg22692158 | cg19437319 |
| cg01109219 | cg27611781 |
| cg04717045 | cg05206587 |
| cg02657438 | cg22024657 |
| cg22959932 | cg24698622 |
| cg25167447 | cg08626653 |
| cg10707565 | cg08176694 |
| cg27015047 | cg04232649 |
| cg14023451 | cg24125648 |
| cg24512973 | cg07498421 |
| cg21137417 | cg04469303 |
| cg08057475 | cg16928795 |
| cg10334928 | cg00954566 |
| cg04457051 | cg17952262 |
| cg02399455 | cg24476569 |
| cg08658594 | cg11953868 |
| cg19589427 | cg09215553 |
| cg17095936 | cg07550362 |
| cg02880679 | cg15827031 |

**Supplementary Table S3.** The performance of different linear and nonlinear ML model including support vector classifier (SVC), Logistic Regression (LR), Decision Tree, Naive Bayes, K-Nearest Neighbors (KNN), and Random Forest

| Dataset | Model | MOFA | MOGCN |
| --- | --- | --- | --- |
| All Omics | SVM | 0.5563 ± 0.0030 | 0.3333 ± 0.0023 |
|  | Logistic Regression | 0.7527 ± 0.0410 | 0.7063 ± 0.0167 |
|  | Decision Tree | 0.6883 ± 0.0385 | 0.6299 ± 0.0355 |
|  | Naive Bayes | 0.4937 ± 0.0837 | 0.5938 ± 0.0542 |
|  | KNN | 0.5754 ± 0.0244 | 0.5423 ± 0.0379 |
|  | Extra Trees | 0.5528 ± 0.0107 | 0.3891 ± 0.0136 |
|  | Random Forest | 0.6323 ± 0.0126 | 0.5721 ± 0.0134 |
| Transcriptomics | SVM | 0.5508 ± 0.0077 | 0.3333 ± 0.0023 |
|  | Logistic Regression | 0.6138 ± 0.0406 | 0.6437 ± 0.0191 |
|  | Decision Tree | 0.6462 ± 0.0276 | 0.6303 ± 0.0389 |
|  | Naive Bayes | 0.0842 | 0.5118 ± 0.0361 |
|  | KNN | 0.6272 ± 0.0406 | 0.5722 ± 0.0212 |
|  | Extra Trees | 0.5532 ± 0.0055 | 0.3888 ± 0.0355 |
|  | Random Forest | 0.6567 ± 0.0316 | 0.6068 ± 0.0144 |
| Epigenomics | SVM | 0.5386 ± 0.0192 | 0.3333 ± 0.0023 |
|  | Logistic Regression | 0.6883 ± 0.0143 | 0.6030 ± 0.0373 |
|  | Decision Tree | 0.6359 ± 0.0229 | 0.5186 ± 0.0452 |
|  | Naive Bayes | 0.5644 ± 0.0359 | 0.5359 ± 0.0201 |
|  | KNN | 0.5990 ± 0.0432 | 0.4340 ± 0.0463 |
|  | Extra Trees | 0.5470 ± 0.0138 | 0.3598 ± 0.0074 |
|  | Random Forest | 0.6110 ± 0.0098 | 0.4409 ± 0.0327 |
| Microbiomics | SVM | 0.3333 ± 0.0023 | 0.3333 ± 0.0023 |
|  | Logistic Regression | 0.5227 ± 0.0293 | 0.6210 ± 0.0215 |
|  | Decision Tree | 0.4375 ± 0.0265 | 0.4959 ± 0.0167 |
|  | Naive Bayes | 0.3931 ± 0.0368 | 0.5732 ± 0.0626 |
|  | KNN | 0.4408 ± 0.0165 | 0.4871 ± 0.0259 |
|  | Extra Trees | 0.3349 ± 0.0066 | 0.3444 ± 0.0084 |
|  | Random Forest | 0.4215 ± 0.0142 | 0.4649 ± 0.0144 |

**Supplementary Table S4.** The pathways enriched from the network of MOFA+ and MoGCN Features.

| MOFA | Hits | FDR | MOGCN | Hits | FDR |
| --- | --- | --- | --- | --- | --- |
| MicroRNAs in cancer | 104 | 1.06E-10 | MicroRNAs in cancer | 52 | 0.000133 |
| Apoptosis - multiple species | 71 | 5.77E-08 | cGMP-PKG signaling pathway | 36 | 6.30E-05 |
| Human immunodeficiency virus 1 infection | 66 | 1.93E-07 | Human immunodeficiency virus 1 infection | 29 | 0.0197 |
| Renal cell carcinoma | 66 | 4.99E-17 | Endometrial cancer | 29 | 6.30E-05 |
| Sphingolipid signaling pathway | 63 | 7.33E-17 | Pathways in cancer | 28 | 0.000191 |
| FoxO signaling pathway | 59 | 8.35E-07 | Yersinia infection | 28 | 6.30E-05 |
| Endometrial cancer | 55 | 9.50E-11 | Chemical carcinogenesis | 27 | 0.000133 |
| cGMP-PKG signaling pathway | 54 | 3.46E-05 | Renal cell carcinoma | 26 | 0.000261 |
| Pathways in cancer | 53 | 8.25E-09 | Colorectal cancer | 25 | 0.000211 |
| Herpes simplex virus 1 infection | 51 | 1.35E-07 | Necroptosis | 25 | 6.11E-06 |
| Yersinia infection | 46 | 4.75E-07 | Human T-cell leukemia virus 1 infection | 24 | 0.000106 |
| Human papillomavirus infection | 46 | 1.31E-10 | Proteoglycans in cancer | 23 | 0.00534 |
| Chemical carcinogenesis | 45 | 1.07E-06 | Hematopoietic cell lineage | 23 | 0.000714 |
| Transcriptional misregulation in cancer | 45 | 1.28E-07 | Transcriptional misregulation in cancer | 22 | 0.00238 |
| Proteoglycans in cancer | 43 | 2.51E-05 | Human papillomavirus infection | 21 | 0.000616 |
| Gap junction | 43 | 5.50E-06 | Adherens junction | 21 | 0.000586 |
| Leukocyte transendothelial migration | 43 | 2.75E-15 | PD-L1 expression and PD-1 checkpoint pathway in cancer | 21 | 0.000337 |
| Hematopoietic cell lineage | 39 | 1.71E-05 | AMPK signaling pathway | 20 | 0.123 |
| Human T-cell leukemia virus 1 infection | 39 | 1.03E-06 | Herpes simplex virus 1 infection | 20 | 0.0533 |
| Kaposi sarcoma-associated herpesvirus infection | 39 | 1.49E-08 | Antigen processing and presentation | 20 | 0.00427 |
| Phospholipase D signaling pathway | 38 | 8.50E-12 | Thermogenesis | 20 | 6.30E-05 |
| Toxoplasmosis | 37 | 3.39E-09 | Progesterone-mediated oocyte maturation | 19 | 0.0583 |
| Colorectal cancer | 36 | 0.000298 | Gap junction | 19 | 0.0353 |
| Epstein-Barr virus infection | 36 | 4.71E-05 | Asthma | 19 | 0.00799 |
| Notch signaling pathway | 36 | 1.52E-05 | Notch signaling pathway | 19 | 0.00534 |
| Phosphatidylinositol signaling system | 36 | 8.48E-11 | Autophagy - animal | 19 | 0.000857 |
| Rheumatoid arthritis | 35 | 9.88E-12 | SNARE interactions in vesicular transport | 19 | 0.000329 |
| Influenza A | 34 | 0.000625 | Leukocyte transendothelial migration | 19 | 6.30E-05 |
| Necroptosis | 34 | 2.48E-06 | Autoimmune thyroid disease | 17 | 0.0111 |
| B cell receptor signaling pathway | 34 | 1.02E-06 | Cell adhesion molecules (CAMs) | 17 | 0.00309 |
| Insulin resistance | 34 | 7.20E-07 | Phospholipase D signaling pathway | 17 | 0.000318 |
| GABAergic synapse | 34 | 1.06E-07 | VEGF signaling pathway | 16 | 0.0394 |
| TNF signaling pathway | 34 | 7.24E-09 | Influenza A | 15 | 0.146 |
| Aldosterone-regulated sodium reabsorption | 34 | 1.22E-09 | Longevity regulating pathway - multiple species | 15 | 0.0541 |
| Asthma | 33 | 0.000415 | Kaposi sarcoma-associated herpesvirus infection | 15 | 0.0277 |
| Wnt signaling pathway | 33 | 0.000215 | Nucleotide excision repair | 15 | 0.0239 |
| Thermogenesis | 32 | 2.50E-07 | Toxoplasmosis | 15 | 0.00896 |
| Shigellosis | 31 | 0.0073 | Ribosome | 15 | 0.0016 |
| Antigen processing and presentation | 31 | 0.00201 | DNA replication | 14 | 0.153 |
| Toll-like receptor signaling pathway | 31 | 7.43E-05 | Apoptosis | 14 | 0.101 |
| Cell adhesion molecules (CAMs) | 31 | 1.52E-05 | Toll-like receptor signaling pathway | 14 | 0.0566 |
| Staphylococcus aureus infection | 31 | 1.01E-07 | Adipocytokine signaling pathway | 14 | 0.0524 |
| Fc gamma R-mediated phagocytosis | 31 | 7.98E-09 | Cell cycle | 14 | 0.0372 |
| Adherens junction | 30 | 0.000932 | Cell cycle | 14 | 0.0197 |
| Complement and coagulation cascades | 30 | 0.000415 | TNF signaling pathway | 14 | 0.00848 |
| Adipocytokine signaling pathway | 30 | 0.00014 | JAK-STAT signaling pathway | 14 | 0.00744 |
| Peroxisome | 30 | 0.000124 | Th1 and Th2 cell differentiation | 14 | 0.00744 |
| Th1 and Th2 cell differentiation | 30 | 5.09E-07 | Basal cell carcinoma | 14 | 0.000452 |
| Central carbon metabolism in cancer | 30 | 3.02E-08 | Renin secretion | 13 | 0.0394 |
| Intestinal immune network for IgA production | 30 | 1.83E-09 | Hepatitis B | 13 | 0.0277 |
| cAMP signaling pathway | 29 | 1.79E-08 | Chronic myeloid leukemia | 13 | 0.00946 |
| Hepatocellular carcinoma | 29 | 1.25E-09 | Fc epsilon RI signaling pathway | 13 | 0.00799 |
| VEGF signaling pathway | 28 | 0.00667 | Hepatocellular carcinoma | 13 | 0.00148 |
| Apoptosis | 28 | 0.0032 | mRNA surveillance pathway | 13 | 0.001 |
| PD-L1 expression and PD-1 checkpoint pathway in cancer | 28 | 0.00201 | Phagosome | 13 | 0.00037 |
| SNARE interactions in vesicular transport | 28 | 0.000126 | Peroxisome | 12 | 0.163 |
| Vasopressin-regulated water reabsorption | 28 | 5.91E-05 | Autophagy - other | 12 | 0.123 |
| Fc epsilon RI signaling pathway | 28 | 6.50E-07 | GABAergic synapse | 12 | 0.0807 |
| Platelet activation | 28 | 1.22E-09 | Staphylococcus aureus infection | 12 | 0.0325 |
| Th17 cell differentiation | 27 | 0.0145 | Prostate cancer | 12 | 0.00989 |
| Autoimmune thyroid disease | 27 | 0.0048 | Morphine addiction | 12 | 2.00E-04 |
| Hepatitis B | 27 | 5.34E-05 | Central carbon metabolism in cancer | 11 | 0.0372 |
| Estrogen signaling pathway | 26 | 0.00308 | Rheumatoid arthritis | 11 | 0.032 |
| Cell cycle | 26 | 0.00173 | cAMP signaling pathway | 11 | 0.0241 |
| p53 signaling pathway | 26 | 0.00038 | Mitophagy - animal | 11 | 0.00816 |
| Type I diabetes mellitus | 26 | 7.61E-05 | Platelet activation | 11 | 0.00799 |
| Measles | 26 | 2.97E-05 | Aldosterone synthesis and secretion | 11 | 0.00699 |
| Chronic myeloid leukemia | 26 | 1.43E-05 | Melanoma | 11 | 0.00178 |
| TGF-beta signaling pathway | 25 | 0.0173 | Breast cancer | 11 | 0.000147 |
| Ribosome | 25 | 4.13E-05 | Prolactin signaling pathway | 10 | 0.0869 |
| Prostate cancer | 24 | 1.52E-05 | Spliceosome | 10 | 0.0776 |
| Renin secretion | 23 | 0.0045 | Choline metabolism in cancer | 10 | 0.0125 |
| T cell receptor signaling pathway | 23 | 0.000307 | Small cell lung cancer | 10 | 0.00946 |
| RIG-I-like receptor signaling pathway | 22 | 0.0146 | Fc gamma R-mediated phagocytosis | 9 | 0.157 |
| Long-term potentiation | 22 | 0.00451 | Phototransduction | 9 | 0.143 |
| JAK-STAT signaling pathway | 22 | 0.00184 | Thyroid cancer | 9 | 0.0412 |
| Ribosome biogenesis in eukaryotes | 22 | 3.34E-05 | Leishmaniasis | 9 | 0.0368 |
| Basal cell carcinoma | 22 | 1.91E-05 | Malaria | 9 | 0.0135 |
| Legionellosis | 21 | 1.78E-05 | Cytosolic DNA-sensing pathway | 8 | 0.157 |
| Neuroactive ligand-receptor interaction | 20 | 0.0145 | African trypanosomiasis | 8 | 0.144 |
| Circadian entrainment | 20 | 0.00244 | Amoebiasis | 8 | 0.144 |
| Arrhythmogenic right ventricular cardiomyopathy (ARVC) | 20 | 0.00244 | Bladder cancer | 8 | 0.142 |
| Renin-angiotensin system | 20 | 0.00127 | Regulation of lipolysis in adipocytes | 8 | 0.135 |
| Bladder cancer | 20 | 0.000144 | Non-small cell lung cancer | 8 | 0.123 |
| Thyroid cancer | 20 | 4.27E-05 | Natural killer cell mediated cytotoxicity | 8 | 0.109 |
| Circadian rhythm | 20 | 3.46E-05 | IL-17 signaling pathway | 8 | 0.0649 |
| Gastric cancer | 20 | 2.90E-05 | Calcium signaling pathway | 8 | 0.0325 |
| Choline metabolism in cancer | 20 | 2.43E-05 | Ras signaling pathway | 8 | 0.00799 |
| Phagosome | 20 | 1.91E-05 | Primary immunodeficiency | 7 | 0.163 |
| Oxytocin signaling pathway | 19 | 0.0138 | MAPK signaling pathway | 7 | 0.0456 |
| Signaling pathways regulating pluripotency of stem cells | 19 | 0.00306 | Vascular smooth muscle contraction | 7 | 0.0239 |
| Amoebiasis | 19 | 0.000505 | Base excision repair | 6 | 0.0453 |
| Salmonella infection | 19 | 0.000299 | Adrenergic signaling in cardiomyocytes | 6 | 0.0299 |
| Melanoma | 19 | 1.42E-05 | Acute myeloid leukemia | 5 | 0.144 |
| Glucagon signaling pathway | 18 | 0.000507 | Alanine, aspartate and glutamate metabolism | 5 | 0.135 |
| Malaria | 18 | 2.82E-05 | ErbB signaling pathway | 4 | 0.115 |
| Cellular senescence | 17 | 0.0173 | Glycosphingolipid biosynthesis - globo and isoglobo series | 3 | 0.143 |
| Chagas disease (American trypanosomiasis) | 17 | 0.00623 | Glycosaminoglycan biosynthesis - keratan sulfate | 3 | 0.126 |
| Cytosolic DNA-sensing pathway | 17 | 0.00475 |  |  |  |
| African trypanosomiasis | 17 | 0.0036 |  |  |  |
| Non-small cell lung cancer | 17 | 0.00201 |  |  |  |
| Leishmaniasis | 17 | 0.000906 |  |  |  |
| Primary immunodeficiency | 16 | 0.00186 |  |  |  |
| Aldosterone synthesis and secretion | 15 | 0.00836 |  |  |  |
| Inflammatory bowel disease (IBD) | 15 | 0.00836 |  |  |  |
| Focal adhesion | 15 | 0.00189 |  |  |  |
| Synaptic vesicle cycle | 15 | 0.000239 |  |  |  |
| Breast cancer | 15 | 2.88E-05 |  |  |  |
| Vibrio cholerae infection | 14 | 0.0175 |  |  |  |
| Cholinergic synapse | 14 | 0.0157 |  |  |  |
| IL-17 signaling pathway | 14 | 0.00916 |  |  |  |
| Cardiac muscle contraction | 14 | 0.00806 |  |  |  |
| Fluid shear stress and atherosclerosis | 13 | 0.000212 |  |  |  |
| Viral myocarditis | 12 | 0.00178 |  |  |  |
| Proximal tubule bicarbonate reclamation | 11 | 0.00806 |  |  |  |
| Dilated cardiomyopathy (DCM) | 11 | 0.00306 |  |  |  |
| Acute myeloid leukemia | 10 | 0.00806 |  |  |  |
| Lysosome | 9 | 0.0145 |  |  |  |
| Rap1 signaling pathway | 5 | 0.0175 |  |  |  |

**Supplementary Table S5.** Subtype-wise F1 score enhancing comparison between single-omics (transcriptomic-only) and multi-omics (MOFA+ and MoGCN) models using logistic regression.

|  | MOFA | MoGCN |
| --- | --- | --- |
| HER2 | 32% | 27.6% |
| Lum B | 37.9 % | 15.5% |
